# Equol: a metabolite of gut microbiota with potential antitumor effects

**DOI:** 10.1186/s13099-024-00625-9

**Published:** 2024-07-07

**Authors:** Jing Lv, Shengkai Jin, Yuwei Zhang, Yuhua Zhou, Menglu Li, Ninghan Feng

**Affiliations:** 1 Wuxi School of Medicine, Jiangnan University, Wuxi, China; 2 Nantong University Medical School, Nantong, China; 3 Department of Urology, Jiangnan University Medical Center, Wuxi, China; 4 Jiangnan University Medical Center, 68 Zhongshan Road, Wuxi, Jiangsu 214002 China

**Keywords:** Equol, Isoflavones, Gut microbiota, Cancer, Estrogen receptors

## Abstract

An increasing number of studies have shown that the consumption of soybeans and soybeans products is beneficial to human health, and the biological activity of soy products may be attributed to the presence of Soy Isoflavones (SI) in soybeans. In the intestinal tracts of humans and animals, certain specific bacteria can metabolize soy isoflavones into equol. Equol has a similar chemical structure to endogenous estradiol in the human body, which can bind with estrogen receptors and exert weak estrogen effects. Therefore, equol plays an important role in the occurrence and development of a variety of hormone-dependent malignancies such as breast cancer and prostate cancer. Despite the numerous health benefits of equol for humans, only 30-50% of the population can metabolize soy isoflavones into equol, with individual variation in gut microbiota being the main reason. This article provides an overview of the relevant gut microbiota involved in the synthesis of equol and its anti-tumor effects in various types of cancer. It also summarizes the molecular mechanisms underlying its anti-tumor properties, aiming to provide a more reliable theoretical basis for the rational utilization of equol in the field of cancer treatment.

## Introduction

A wealth of evidence suggests that the intake of soybeans and soy products plays a crucial role in human health [1–3]. Soybeans contain various physiologically active substances that are highly beneficial to the body, such as soy isoflavones(SI), soy lecithin, and soy peptides [4]. Research suggests that increased intake of SI is associated with the prevention of various diseases, including a reduced risk of cardiovascular disease [5] and hormone-dependent cancers such as breast and prostate [6, 7], prevention of osteoporosis [8], alleviation of menopausal and depressive symptoms [2], as well as promoting skin health [9]. SI and their partial metabolites have a similar structure to endogenous estrogen 17β-estradiol. They can bind to estrogen receptors (ERs) and exhibit biological activities similar to estrogens; hence, they are referred to as “phytoestrogens” [10]. Previous studies have indicated that phytoestrogens play a crucial role in maintaining human health and may contribute to the prevention and management of various diseases. For instance, feeding infants with soy-based formula milk leads to a significant increase in the concentration of isoflavones in their plasma. Research demonstrated that during the early stages of life, the concentration of isoflavones in plasma is approximately 13,000–22,000 times higher than that of estradiol [11]. This early-life exposure to phytoestrogens may effectively reduce the incidence rate of hormone-dependent diseases in the long term [11]. Additionally, foods or supplements rich in phytoestrogens can improve vasomotor menopausal symptoms (such as hot flashes and night sweats) in perimenopausal and postmenopausal women [12].

Isoflavones in the human body primarily originate from the consumption of soy and soy-derived food products. The isoflavones in soy mainly include genistein, daidzein, and glycitein [13]. However, the SI in soybeans are almost entirely present in the form of glycoside compounds genistin, daidzin and glycitin. The three primary isoflavones and their glycosides make up approximately 50%, 40%, and 10% of the total isoflavone content in soybeans [14]. Isoflavone glycosides are not easily absorbed in the human gastrointestinal tract, resulting in lower bioavailability. In contrast, isoflavone aglycones exhibit a significantly higher bioavailability compared to other types of glycosides [15]. For example, daidzin, presented as a glycoside, is capable of undergoing hydrolysis to daidzein by human β-glycosidases(lactase), which are located in the brush border of the small intestine, as well as by β-glucosidase enzymes secreted by gut microbiota. Subsequently, daidzein is absorbed under the influence of specific gut microbiota. Therefore, in order to enhance the absorption efficiency of SI, it is crucial to convert the glycoside form of SI into their corresponding aglycone forms.

Equol, a metabolite of SI produced by specific gut microbiota, exhibits affinity for estrogen receptors (ERs), including estrogen receptor alpha (ERα) and estrogen receptor beta (ERβ). It functions as an estrogen regulator and plays a beneficial role in various hormone-dependent diseases [16, 17]. These include improving menopausal symptoms, preventing osteoporosis, and reducing the risk of breast and prostate cancer [2, 18–20]. The impact of equol on hormone-dependent tumors may benefit from its binding with ERs, while ERα and ERβ have been proven to play crucial roles in the development of tumors. For instance, ERα is usually upregulated in the early stages of cancer and acts as a promoting factor for tumor growth, while ERβ is downregulated during carcinogenesis and cancer progression, acting as a suppressor of tumor growth [21]. However, the inhibitory effect of ERβ in cancer is not absolute. Increasing evidence suggests that ERβ plays a pro-carcinogenic role in ERα-negative cancers, and its function in tumors highly depends on co-expression with ERα. Many other factors such as co-regulatory factors, other steroid receptors, transcriptional regulators, as well as the presence of endogenous and exogenous ligands can influence the action of ER [22–24]. In addition to binding to ERs, equol can also exhibit strong anti-androgen activity by specifically binding to 5α-dihydrotestosterone and inhibiting its binding to androgen receptors [25]. Furthermore, the antioxidant effect is believed to play a crucial role in inhibiting the occurrence and development of tumors [26]. Research has shown that equol, a metabolite of SI, exhibits the strongest antioxidant activity among its counterparts. It can inhibit oxidative stress damage, promote the expression of antioxidant genes in cells, and enhance the activity of antioxidant enzymes [27–29]. Studies have reported that equol can exert anti-tumor effects by inhibiting the activation of important transcription factor AP-1 in the MEK signaling pathway-induced cell transformation [30]. In summary, equol plays an extremely important role in promoting human health, particularly in tumor suppression. However, current research on the synthesis and metabolism of equol within the human body as well as its mechanisms of action in various types of tumors remains insufficient and warrants further investigation.

This review primarily describes the chemical properties and metabolism of equol, summarizes the current research on intestinal microbiota associated with equol synthesis, and discusses its role in hormone-dependent and non-hormone-dependent tumors. It provides a reference for further understanding the relationship between individual differences in equol and diet or intestinal microbiota, as well as guiding subsequent studies on the preventive effects of equol in various types of tumors and its underlying mechanisms.

## Biological properties of equol

Equol was first isolated from the urine of pregnant mares in 1932, subsequently identified as an isoflavonoid, and detected in human urine and blood 50 years later [31]. Equol is a degradation product of soy glycosides formed by specific gut microbiota in the human digestive tract, and due to the presence of a chiral carbon at the C-3 position of its furanoid ring, equol exists as both R- and S- isomers [31]. However, only S-equol is produced by the metabolism of soybean glycosides by gut microbiota, whereas the synthetic compound racemic equol is a mixture of S-equol and R-equol [32]. The two isomers of equol exhibit different binding affinities to ERα and ERβ. S-equol has a 13-fold higher affinity for ERβ compared to ERα, while R-equol exhibits stronger binding to ERα, being 4-fold than that of S-equol [33]. These findings are important for understanding the role of equol in the human body and its potential health effects.

**Fig. 1:**
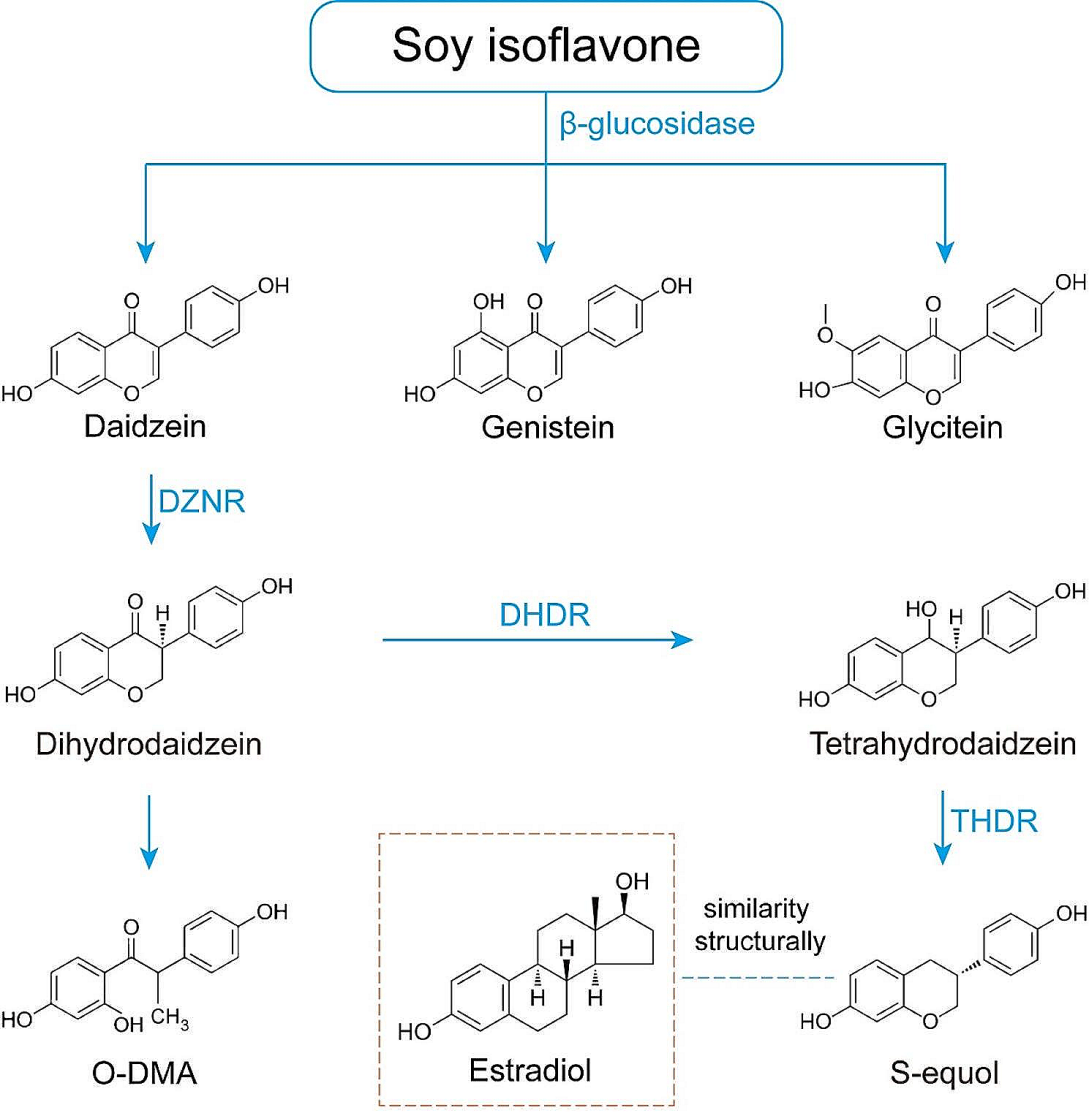
Diagrammatic representation of equol biosynthesis and the similarity of equol to estrogen. DZNR: Daidzein Reductase; DHDR: Dihydrodaidzein Reductase; THDR: Tetrahydrodaidzein Reductase

The conversion of daidzein to equol is a complex process that requires the involvement of gut microbiota and specific enzymes they produce. Daidzein is first metabolized in the intestine to dihydrosoyosides (DHD) and tetrahydrosoyosides (THD), and finally converted to S-equol and O-desmethylangolensin (O-DMA) (which has received less attention due to its lack of significant biological activity) [34].Shimada et al. purified a novel NADP(H)-dependent L-daidzein reductase (L-DZNR) from the lactic acid bacterium Lactococcus 20–92 and elucidated the mechanism by which recombinant histidine-tagged L-DZNR converts soybean glycoside to enantioselective (S)-DHD [35]. In another of their studies, they identified three other key enzymes, L-dihydrodaidzein reductase (L-DHDR), L-tetrahydrodaidzein reductase (L-THDR), and L-dihydrodaidzein racemase (L-DDRC) (which are associated with increased synthesis of equol) and elucidated the metabolism of daidzein to S-equol by these key enzymes [36, 37] (as shown in Fig. 1).

Compared to other flavonoid metabolites, equol is more stable and exists in a higher proportion as a free form in the human body [38]. This means that the concentration of equol in the blood is higher, and it can exert its antioxidant and other potential health benefits for a longer period of time in the body. However, not everyone can benefit from it because there are significant differences in the ability of individuals to produce equol. Only about 30% of the Western populations and 60% of the Asian populations can produce equol [39]. Due to individual differences in intestinal microbiota, only approximately 25–50% of the global population can metabolize daidzein into equol. These individuals are referred to as equol producers (EP), while those who cannot produce equol are called equol non-producers (ENP) [40]. Research has shown that the proportion of EP is significantly higher among vegetarians compared to NEP [41]. This suggests that different dietary components may affect the synthesis of equol by influencing the abundance of specific gut microbiota. Although equol is a metabolite of SI in the human body, increasing intake of SI does not elevate equol concentrations for NEP. Studies have reported that most NEP metabolize SI into O-DMA, which lacks estrogenic activity [42]. Védrine et al. found that postmenopausal women defined as NEP did not experience an increase in their internal levels of equol after consuming 100 mg/day of SI for one month [43]. Liang et al., through treatment with broad-spectrum antibiotics on mice for three weeks, established a Pseudo Germ-Free Mice model. They then transplanted gut microbiota from equol-producing individuals into these mice and observed a significant enhancement in their ability to produce equol [44].In conclusion, although many factors such as diet habits, age, gender, and genetic factors can influence the absorption of equol; due to its decisive role in metabolizing SI, the production level primarily depends on whether there exists gut microbiota involved in synthesizing equol within an individual’s body [45, 46].

## Equol and gut microbiota

Human intestines are colonized by a large number of microorganisms, mainly bacteria, forming a highly complex ecosystem known as the “gut microbiota“ [47]. The metabolic capacity of the gut microbiota is approximately 100 times that of the human liver, thus playing an important role in the metabolism of human diet [48]. For humans, there is significant variation in the ability to produce equol among individuals, with only about 25–50% of the global population able to metabolize daidzein into equol. Additionally, Western populations have a much lower proportion of equol producers compared to Asian populations [39, 46]. The ability to produce equol in the human body primarily depends on whether there is an equol-metabolizing microbial community present in the gut. Some studies suggest that individuals who are equol producers have a lower risk of hormone-dependent diseases compared to non-equol producers [49, 50]. Consequently, discovering gut strains capable of metabolizing and producing equol holds significant importance for human health and warrants further investigation.

With further research, scientists have discovered bacteria that can participate in the biosynthesis of equol, which is mainly found in the gut microbiota [51] (as shown in Table 1). Early studies found that equol was undetectable in the bodies of newborns, and equol was also undetectable in the urine of germ-free animals fed with soy. However, when human fecal samples were co-cultured with daidzein from soy, both dihydrodaidzein and equol could be detected [52–54]. Further investigations involved co-culturing fecal samples from EP and non-producers with daidzein under anaerobic conditions at 37℃ for five days. The results showed that daidzein was converted into equol when co-cultured with fecal samples from EP, but not with NEP. Additionally, there were significant differences in subjects’ metabolism of daidzein in the presence of antibiotics. Antibiotics inhibited equol production but had no effect on DHD production [55]. Research has found that children who consume soy-based foods experience a decrease in urinary equol levels after oral antibiotic administration, compared to before taking antibiotics [56]. This further demonstrates the impact of antibiotic intervention on equol production, which is closely associated with gut microbiota. While gut microbiota break down SI to produce equol, only S-equol is formed by gut microbiota [32]. Currently, our understanding of the microbial communities involved in the breakdown and metabolism of equol remains limited, necessitating further research to explore this complex relationship between equol and gut microbiota.

**Table 1:** Related strains involved in the metabolism transformation of equol and its intermediate precursors

Origins	Substrate	Product	Bacterial strains	Classifications	References
Non-Independent Equol-Producing Bacteria					
Human	DHD	Equol	Julong732	*Eggerthella*	[57]
Human	DHD	Equol	FJC-A10	*Adlercreutzia equolifaciens*	[58]
Human	DHD	Equol	FJC-A161	*Adlercreutzia equolifaciens*	[58]
Human	Daidzein	DHD	HGH6	*Clostridium*	[59]
Human	Daidzin	Daidzein	HGH21	*Escherichia coli*	[59]
Human	Daidzin	Daidzein	MRG-1	*Coprobacillus*	[60]
Human	Daidzein	DHD	TM-40	*Coprobacillus*	[61]
Human	Daidzein	DHD	INIA P333	*Enterococcus*	[62]
Human	Daidzein	DHD	INIA P540	*Lactobacillus*	[62]
Human	Daidzin	Daidzein	*Bifidobacterium* MB	*Bifidobacterium*	[63]
Human	Daidzein	DHD	HXBM408	*Pediococcus acidilactici*	[64]
Bovine	Daidzein	DHD	Niu-O16	*Lactobacillus*	[65]
Independent Equol-Producing Bacteria					
Human	Daidzein/ DHD	Equol	YY7918	*Eggerthella*	[66]
Human	Daidzein	Equol	*Lactococcus* 20–92	*Lactococcus*	[67]
Human	Daidzein	Equol	FJC-B9T	*Adlercreutzia equolifaciens*	[58]
Human	Daidzein	Equol	ATCC15700	*Bifidobacterium breve*	[68]
Human	Daidzein	Equol	BB536	*Bifidobacterium longum*	[68]
Human	Daidzein	Equol	TM-30	*Coriobacteriaceae*	[69]
Human	Daidzein	Equol	CS1 CS2 CS3	*Pediococcus* *Lactobacillus* *Lactobacillus*	[70]
Human	Daidzein	Equol	Y11	*Slackia equolifaciens*	[71]
Rat	Daidzein	Equol	AHU1763	*Asaccharobacter celatus*	[72]
Rat	Daidzein	Equol	do03	*Asaccharobacter celatus*	[73]
Rat	Daidzein	Equol	LH-52	*Proteus mirabilis*	[74]
Mouse	Daidzein	Equol	MT1B8	*Enterorhabdus mucosicola*	[75]
Mouse	Daidzein	Equol	JCM1123(T)	*Lactobacillus collinoides*	[76]
Rat	Daidzein	Equol	JCM 7548	*Lactobacillus intestinalis*	[77]
Pig	Daidzein	Equol	D1,D2	*Eubacterium*	[78]

### Non-independent equol-producing bacteria

Current research reports that some gut microbiota metabolize daidzein, a compound found in soybeans, into intermediate products such as dihydrodaidzein (DHD) and tetrahydrodaidzein (THD), rather than catalyze the full conversion from daidzein to equol. However, other gut microbiota can only utilize these intermediate metabolites to synthesize equol. These bacteria, participating in specific stages of the equol metabolism process, are known as non-independent producers of equol [65]. The human intestinal anaerobic bacterium *Eggerthella* sp. Julong 732 is the first reported bacterium that can produce equol non-independently. It is a rod-shaped, Gram-negative anaerobic bacterium isolated from human feces, and it can only metabolize DHD and THD into equol [57]. The discovery of the Julong 732 strain has provided the first evidence that a single gut bacterium community can metabolize DHD into S-equol. Maruo et al. also isolated two Gram-positive cocci bacteria, *Eubacterium* FJC-A10 and FJC-A161, from human feces, which can only metabolize DHD into equol [58]. Hur et al. screened a Gram-negative bacterial *Clostridium* sp. strain HGH6 from healthy individuals’ feces, capable of metabolizing daidzein and genistein into dihydrodaidzein and dihydrogenistein under anaerobic conditions [59]. Additionally, strains such as *Escherichia coli* HGH21 [59], *Coprobacillus* sp. MRG-1 [60], *Coprobacillus* sp. TM-40 [61], *Enterococcus faecalis* INIA P333 [62], *Lactobacillus rhamnosus* INIA P540 [62], and *Bifidobacterium* MB [63] also exhibit similar functions. In addition to human commensal bacteria, Xie et al. isolated a facultative anaerobic Gram-positive bacterium, *Pediococcus acidilactici* HXBM408, from fresh feces of pregnant mares, which is capable of converting genistin to dihydrogenistein [64]. It is interesting to note that researchers attempted to co-cultivate two different strains of bacteria, Julong 732 (which can only metabolize daidzein into DHD) and *Lactobacillu*s sp. Niu-O16 (which can only metabolize DHD into S-equol), together with daidzein as the substrate. They found that almost all the daidzein was converted into equol [65]. This suggests that non-independent equol-producing bacteria can participate in the synthesis of equol through synergistic interactions.

### Independent equol-producing bacteria

Recent studies reported bacterial isolates from both humans and certain animals, predominantly belonging to the Coriobacteriaceae family, that are capable of independently producing equol. Yokoyama et al. isolated a Gram-positive anaerobic rod-shaped bacterium, *Eggerthella* sp. YY7918, from healthy individuals’ feces, capable of converting daidzin and dihydrodaidzin into equol [66]. Uchiyama et al. also isolated a lactic acid bacterium, *Lactococcus garvieae* 20–92, from human feces that produces equol. This is the first report of a lactic acid bacterium directly metabolizing daidzin into equol [67]. In addition, there are strains of bacteria isolated from the human body such as *Adlercreutzia equolifaciens* FJC-B9 [58], *Bifidobacterium breve* ATCC15700 and *Bifidobacterium longum* BB536 [68], *Slackia* TM-30 [69], *Pediococcus pentosaceus* CS1, *Lactobacillus paracasei* CS2, *Lactobacillus sakei/graminis* CS3 [70] and *Slackia equiolifaciens* Y11 [71]. Minamida et al. first isolated a strain of Gram-positive, rod-shaped anaerobic bacteria *Asaccharobacter celatus* AHU1763 from the rat intestine, which is capable of metabolizing daidzein into equol [72]. In addition, other rodent-derived bacteria that produce equol include *Asaccharobacter celatus* do03 [73], *Proteus mirabilis* LH-52 [74], *Enterorhabdus mucosicola* MT1B8 [75], *Lactobacillus collinoides* JCM1123(T) [76], and *Lactobacillus intestinalis* JCM 7548 [77]. Yu et al. isolated two strains of equol-producing bacteria, *Eubacterium* D1 and D2, from pig feces, providing the first report on the presence of daidzein-metabolizing bacteria in mammalian intestines [78].

The discovery of bacteria that produce equol independently directly confirms the relationship between intestinal microbiota and equol metabolism. In recent years, with the increasing discovery of equol-producing bacteria, researchers have also isolated such bacteria from certain foods. For example, Gram-positive rod-shaped anaerobic bacterial strains SNR45DH-1 and SNR48DH-1 (Family Coriobacteriaceae) were isolated from fermented brine of stinky tofu, capable of converting daidzein to S-equol. This is the first report of equol-producing bacteria being isolated from food [79]. The types of bacteria involved in equol metabolism are diverse, and their interactions also affect the efficiency of equol metabolism. Human-derived equol-producing bacteria mainly come from the intestines, with the majority being strict anaerobes and a minority facultative anaerobes. Thus, anaerobic conditions may be crucial for equol metabolism. Additionally, some undigested fermentation products in the colon, such as hydrogen gas, propionate, and butyrate salts can promote the conversion of soy glycosides into equol [54]. Therefore, further exploration of conditions for equol production is a key area for research that can aid in proper dietary adjustments and enhancing the biosynthesis efficiency of equol.

## The anti-tumor effect of equol

An increasing number of studies have shown that SI and their metabolites have potential preventive effects on various types of tumors, such as breast cancer and prostate cancer [16]. Epidemiological research has found that the incidence rates of breast, prostate, and colorectal cancers are lower in Asian populations compared to Western populations. This difference may be attributed to dietary habits and genetic factors, as Asian diets are rich in high levels of isoflavones [80–82]. The daily intake of SI in Finland and the UK is 0.8 mg and 7-9 mg respectively, while it is 97 mg in China and 39.5 mg in Japan [83–86]. Studies have indicated that the anti-tumor effect of SI mainly depends on their metabolite equol. Equol exhibits the highest affinity for ERs among all metabolites and demonstrates strong anti-tumor effects in multiple tumor models by inhibiting tumor occurrence, development, metastasis, and prognosis [87] (as shown in Table 2). Furthermore, equol can be used in combination with some common chemotherapy drugs to enhance their efficacy against cancer while reducing side effects caused by chemotherapy drugs [87, 88].

**Table 2:** Anticancer molecular mechanism of equol

Type of cancer	Cancer model	Effect	Mechanism	Equol concentration	Reference
Breast	MDA-MB-231 MCF-7	DNA demethylating	demethylating of BRCA1 and BRCA2	S-equol 2 μm	[89]
	MCF-7	NA	NA	Equol 0–40 μm	[88]
	MDA-MB-231	Inhibit metastasis	↓MMP-2	R-equol and S-equol(0,2.5,10,50µM)	[90]
	MCF-7	Induces apoptosis	↑Bax/Bcl-xL	Equol 0–100 μm	[91]
	MDA-MB-453	Induces apoptosis Anti-proliferative	↑Caspase-9 ↑cytochrome c	Equol 0–100 μm	[19]
	MDA-MB-231	Induces apoptosis	↓NF-κB	Equol 0–200 μm	[92]
	MCF-7	Induces apoptosis Anti-proliferative	↑miR-10a-5p Inhibit PI3K/AKT pathway	Equol 0-400ug/ml	[93]
	MCF-7	Induces apoptosis	↓bcl-2/bax ↑caspase-9 and caspase-7 ↑cytochrome c	Equol 0–100 μm	[94]
	Mouse model	Induces apoptosis	↑Caspase-3	Equol (2.7 µmol)/kg	[95]
prostate	PC-3	Anti-proliferative	↑ERRγ	Equol 0–20 μm, 4mM	[96]
	PC-3 LNCaP	Anti-proliferative	↑DNA damage	Equol 0–500 μm	[97]
	DU145	Inhibit metastasis	↓MMP-2 ↓MMP-9 ↓u-PA	Equol 0–50 μm	[98]
	PC-3 LNCaP DU145 Mouse model	G2/M arrest Induces apoptosis	↓Cyclin B1 ↓CDK1 ↑p21 and p27 ↑FasL and Bim ↑FOXO3a ↓p-FOXO3a ↓MDM2	Equol 0–200 μm	[20]
	LNCaP CxR 22Rv1	Anti-proliferative	↑Skp2 ↓AR	Equol 0–50 μm	[99]
Osteosarcoma	SKOV3 BG1 ES2 SKOV3-TR	Anti-proliferative Inhibit metastasis Induces apoptosis	↑caspase 3 ↑caspase 7	S-equol 0–200 μm	[100]
Hepatocellular	SMMC-7721	Anti-proliferative S-phase arrest Induces apoptosis	↑caspase 12 ↑caspase 8 ↑Chop and Bip ↑caspase 3 ↑Bax ↓Bcl-2	S-equol 0-100umol/L	[101]
	HepG2	Anti-proliferative Inhibit metastasis	↓hexokinase ↓phosphofructokinase ↓pyruvate kinase M2	(-)-5-hydroxy-equol 0–40 μm	[102]
Gastric	MGC-803	G1/G0 arrest Induces apoptosis	↓Cyclin D1 ↓Cyclin E1 ↓CDK2 ↓CDK4 ↑P21 ↑caspase 3	Equol 0-80umol/L	[103]
	MGC-803	Anti-proliferative Induces apoptosis	↓cIAP1 ↓Bcl-xL ↓Bid ↑caspase 3 ↑caspase 9	Equol 0–20 μm	[104]
Colon	HCT-15	Anti-proliferative	↑ERβ ↑Nrf2	(±)-Equol R-Equol 0-10umol/L	[105]
	HCT-15 LOVO SW480	Anti-proliferative	↑ERβ ↑Nrf2	Equol 0-10umol/L	[106]
Lung	Mouse model	Inhibited Tumor Growth	↑Nrf2	(±)-Equol S-Equol R-Equol 250 mg/kg and 500 mg/kg	[107]
	HCC827 H1993	Anti-proliferative	NA	Equol 0–100 μm	[108]
Cervix	HeLa	Induces apoptosis	↑caspase 3 ↑caspase 8 ↑caspase 9 ↓Bid	Equol 0–20 μm	[109]
Pancreatic	Su 86.86	Anti-proliferative	↓K-ras	Equol 0–10 μm	[110]

### Inhibitory effect of equol on hormone-dependent tumors

#### Breast cancer

Breast cancer is the fourth leading cause of cancer-related deaths worldwide and a major cause of death among women [111]. It is an hormone-dependent tumor, and increasing evidence suggests that endogenous estrogen plays a crucial role in its development. Elevated levels of endogenous estrogen are considered one of the mechanisms for breast cancer incidence [111]. Equol, due to its similar chemical structure to 17-β estradiol, exhibits estrogenic activity and is known for its classic biological activity as an estrogen-like compound [112]. Depending on the levels of endogenous estrogens in the body, equol can act as either an agonist or antagonist. It can bind to the ER and exert weak estrogenic effects or competitively bind with estradiol on nuclear ERs to exhibit antiestrogenic effects, thereby maintaining stable hormone levels within the body [113]. In a case-control study conducted by Lee et al., it was reported for the first time that soy products could reduce the risk of premenopausal women developing breast cancer. This may be attributed to the presence of phytoestrogens in soy products [114]. A prospective study from Japan also found a correlation between regular consumption of foods rich in isoflavones and reduced risk of breast cancer incidence [6]. An epidemiological survey discovered a positive correlation between increased urinary equol levels in premenopausal women and decreased risk of breast cancer [115]. Furthermore, early exposure to soy products has been shown to lower breast tumor incidence. Several case-control studies have confirmed a significant negative association between childhood or adolescent soy intake and risk of developing breast cancer [116–118]. However, several nested case-control studies have found no significant association between soy isoflavone intake and breast cancer risk in Western populations [119, 120]. These epidemiological contradictory findings may be attributed to the earlier studies’ failure to distinguish between equol producers and non-producers. This is because the anti-tumor effects of SI primarily depend on their metabolite equol, which can only be produced by approximately 30% of Western populations and 60% of Asian populations [39, 87].

Equol’s estrogenic activity has been extensively demonstrated in many in vitro studies to inhibit the progression of breast cancer. It achieves this by binding to ERα and ERβ, inhibiting the proliferation, migration, and invasion of breast cancer cells, as well as inducing apoptosis [121]. Eun et al. found that equol suppresses tumor cell proliferation by inducing apoptosis mediated by caspase activation cascade in breast cancer cells [19]. S-equol can also induce CpG island demethylation within the BRCA1 and BRCA2 gene promoters in MDA-MB-231 and MCF-7 breast cancer cell lines, resulting in tumor inhibition [89]. However, there are also reports suggesting that low concentrations of equol can activate ERK1/2 to induce entry into the S phase for MCF-7 breast cancer cells, promoting tumor cell proliferation [122]. Hatono et al. demonstrated a dual effect of equol on estrogen receptor-positive breast cancer cell lines: at low doses it promotes cell growth while at high doses it exhibits anti-tumor effects. Furthermore, when used in combination with tamoxifen (TMX), at low concentrations equol competitively binds to ERα inhibiting TMX’s anti-tumor effects; whereas at high concentrations a synergistic anti-tumor effect is observed [122]. Therefore, it is evident that different concentrations of equol have inconsistent effects on breast cancer cells and its actions vary between ER-positive and ER-negative subtypes. Matrix metalloproteinases (MMPs) play a crucial role in tumor migration and invasion processes. Studies have reported that both R-equol and S-equol can inhibit the invasion of human breast cancer MDA-MB-231 cells by downregulating matrix metalloproteinase 2 (MMP-2) [90].

Equol can induce apoptosis in breast cancer cells, thereby inhibiting tumor growth. Studies have reported that equol induces tumor cell apoptosis by upregulating Bax/Bcl-xl expression [91] and increasing the release of cytochrome c [19]. Additionally, equol can induce apoptosis in human breast cancer MDA-MB-231 cells by inhibiting NF-κB expression [92]. Zhang et al. found that compared to normal breast epithelial cells MCF-10 A, S-equol inhibits the proliferation and promotes apoptosis of breast cancer cells MCF-7 by upregulating miR-10a-5p and inhibiting the PI3K/AKT pathway, while having minimal impact on MCF-10 A cells [93]. Currently, drug resistance is a common phenomenon in cancer treatment, which severely affects the efficacy of anticancer drugs for breast cancer. Therefore, combination therapy has attracted widespread attention in the field of cancer treatment. The combination use of equol with chemotherapy drugs has been studied due to its effective anticancer effects. Tamoxifen (an ERα antagonist) and its active metabolite 4-hydroxytamoxifen (4-OHT) are widely used for prevention and treatment of breast cancer [123, 124]. Christiana et al. investigated the effects of equol and 4-hydroxytamoxifen alone or in combination on hormone-dependent MCF-7 breast cancer cells. They found that compared to individual compounds, combined use of equol (> 50 μm) with 4-hydroxytamoxifen (4-OHT; >100nM) significantly enhanced apoptotic induction while reducing cell viability in MCF-7 breast cancer cells [94]. Furthermore, Liu et al., through in vivo experiments using rats induced with 7,12-dimethylbenz(a)anthracene (DMBA)-induced mammary tumors and xenografts from human MCF-2 breast cancer cells transplanted into ovariectomized athymic nude mice fed with tamoxifen or equimolar doses of genistein or equol, observed potential inhibitory effects on rodent mammary tumor growth by genistein and equol after several weeks; moreover, their inhibitory activity was superior to tamoxifen [95]. In conclusion, these findings demonstrate that equol enhances the chemotherapy effect of tamoxifen. The synergistic interaction between equol and anticancer drugs may open up new avenues for overcoming drug resistance in chemotherapy.

#### Prostate cancer

Prostate cancer is the second most common cancer and the fifth leading cause of death among men worldwide [125]. Epidemiological studies have shown that consumption of soy products and foods rich in plant estrogens can reduce the risk of prostate cancer [7, 126]. Several studies have indicated a significant negative correlation between plasma equol concentration and the incidence of prostate cancer [127, 128]. Another case-control study involving prostate cancer patients from Japan, Korea, and the United States revealed a negative association between the proportion of equol producers and the incidence rate of prostate cancer [129]. However, an epidemiological study found no clear trend between high serum equol concentration in Japanese men and lower risk of developing prostate cancer, while there was no association observed between serum equol concentration and prostate cancer risk among European men [130]. The limitation of this epidemiological study lies in its reliance on testing only blood samples for equol concentration, which may not reflect mid to long-term levels of equol within the body.

In addition to these epidemiological studies, there have been numerous in vitro and in vivo experiments investigating the effects of equol on prostate cancer. Its mechanisms of action include inhibition of proliferation, induction of cell cycle arrest and apoptosis. Johanna et al. found that equol stimulates estrogen-related receptor γ (ERRγ) transcriptional activity, thereby inhibiting the growth of prostate cancer PC-3 cells [96]. Equol significantly inhibited the growth of LNCap cells at concentrations ≥ 10 μm and PC-3 cells at concentrations ≥ 0.1 μm, while causing DNA strand breaks in both cell lines at concentrations > 250 μm [97]. Thus, it appears that equol can inhibit the growth of prostate cancer cells by inducing DNA damage at higher concentrations. In addition, equol can also inhibit invasion of DU145 prostate cancer cells by downregulating matrix metalloproteinase 2 (MMP-2) and MMP-9 [98]. Lu et al. demonstrated that compared to normal prostatic epithelial cells RWPE-1, S-equol and R-equol inhibit the proliferation of prostate cancer cells PC3, DU145, and LnCaP in a concentration-dependent manner, with minimal effect on RWPE-1 cells. This suggests that S- and R-equol have selective activity against malignant cells [20]. Additionally, they also found that S-equol induces apoptosis and cell cycle arrest in prostate cancer PC3 cells by activating AKT/FOXO3a pathway and suppressing MDM2 complex expression (a negative regulator for tumor suppressor p53), as well as inhibiting the growth of PC3 xenografts in BALB/c nude mice. Furthermore, Equol exerts anti-androgenic effects to inhibit progression of prostate cancer. Studies have shown that equol inhibits Skp2-mediated degradation of androgen receptor (AR), thereby suppressing the growth of prostate cancer through S-phase kinase-associated protein 2(Skp2)-mediated AR degradation [99], as well as specifically binding to 5α-dihydrotestosterone to prevent its binding with AR [25].

Numerous studies have confirmed the significant role of a high-fat diet (HFD) in the development of prostate cancer, although its underlying mechanisms remain largely unknown [131]. Liu et al. utilized a transgenic mouse model of prostate cancer (TRAMP) and found that after intervention with HFD or normal diet followed by oral administration of daidzein for four days, mice in the HFD group exhibited significantly decreased serum equol levels. Furthermore, analysis using 16 S rRNA sequencing revealed differences in intestinal microbiota composition between the HFD group and control group, with a decrease in *Adlercreutzia* bacteria abundance, which is responsible for producing equol [132]. These findings suggest that HFD may promote prostate cancer development by inhibiting the growth of equol-producing bacteria. Tanaka et al., on the other hand, supplemented healthy Japanese subjects with SI and observed a significant increase in serum sex hormone-binding globulin levels along with a notable decrease in serum free testosterone and dihydrotestosterone (DHT) levels after three months [133]. Interestingly, among ten non-equol producers who were given soy isoflavone supplementation for three months, two individuals showed detectable production of equol in their serum samples along with reduced DHT levels [133]. These results indicate that short-term intake of SI can stimulate equol production while reducing serum DHT levels. Additionally, long-term and continuous supplementation may potentially convert non-equol producers into producers. Of course, this needs to be confirmed by further studies. The role of Equol in the prevention and treatment of prostate cancer in men has received increasing attention; however, due to factors such as dose, ethnicity, soy food intake, and individual intestinal microbiota specificity, the role of Equol in the development of prostate cancer needs further study.

#### Other hormone-dependent tumors

Ovarian cancer is considered to be a hormone-dependent tumor, as approximately 60-100% of tumors express ERs (ERα and ERβ), with a decrease in ERβ expression observed during tumor progression [134, 135]. A case-control study conducted in Japan showed that decreased levels of serum genistein are associated with an increased risk of epithelial ovarian cancer [136]. Another study demonstrated that genistein inhibits the proliferation, migration, and invasion of human ovarian cancer cells SKOV-3 and A2780CP, induces cell cycle arrest, and promotes apoptosis [137]. Liu et al. found that the ER-β agonist S-equol reduces the viability of ovarian cancer cells, inhibits their migration and invasion, and promotes apoptosis. Furthermore, equol exhibits tumor-suppressive effects in drug-resistant ovarian cancer model cells and sensitizes ovarian cancer cells to cisplatin and paclitaxel treatment [100]. Epidemiological studies have reported an association between intake of soy foods and a reduced risk of endometrial cancer [138]. There is limited research on equol’s tumor-inhibiting effects and mechanisms in hormone-dependent tumors such as ovarian cancer and endometrial cancer; further studies are needed to fully confirm these effects.

### Inhibitory effects of equol on non-hormone-dependent tumors

Due to its dual action as both an estrogen and anti-estrogen, The tumor inhibitory activity of equol is primarily focused on hormone-related tumors. However, recent studies have also found the expression of ER and AR in some non-hormone-dependent tumors [139, 140]. Furthermore, increasing evidence suggests that equol can inhibit the progression of tumors such as colorectal cancer, gastric cancer, non-small cell lung cancer, and liver cancer. This has led to a growing recognition of equol’s role in non-hormone-dependent tumors.

#### Liver cancer

Primary liver cancer is the third leading cause of cancer-related deaths worldwide, with hepatocellular carcinoma (HCC) accounting for 75-95% of cases. The incidence and mortality rates of HCC are increasing annually [141]. Research has shown that the risk of developing HCC in males is 2–5 times higher than in females, suggesting a potential protective role of estrogen in HCC carcinogenesis [142]. Plant-derived estrogen equol can bind to ERs and exert estrogen-like effects, potentially playing a role in the occurrence and progression of liver cancer. Liang et al. found that equol can induce cell cycle arrest at the S phase and exhibit anti-proliferative effects on human liver cancer SMMC-7721 cells. Furthermore, their study reported that equol can induce apoptosis by activating caspase-12, caspase-8, as well as upregulating endoplasmic reticulum stress-associated molecules Chop and Bip, thereby exerting tumor-suppressive effects [101]. Metabolic reprogramming characterized by altered glucose metabolism is a key feature of cancer cells. Targeting tumor cell glucose metabolism represents an effective approach for selectively killing cancer cells [143]. A metabolomics study discovered that (-)-5-hydroxy-equol inhibits the progression of hepatocellular carcinoma by suppressing glycolysis. Researchers found that (-)-5-hydroxy-equol regulates glycolysis in HCC by inhibiting glucokinase, fructokinase, pyruvate kinase activity, as well as downregulating pyruvate kinase M2 expression. This inhibition leads to suppressed proliferation, migration, and invasion of SMMC-7721 cells and inhibited proliferation in HepG2 cells [102].

#### Gastric cancer

Gastric cancer (GC) is the fifth most common malignant tumor worldwide and the fourth leading cause of cancer-related deaths [144]. The incidence rate of GC in males is approximately twice that in females, which may indicate a protective role of sex steroids such as androgens and estrogens in gastric cancer development [145]. A nested case-control study conducted in Korea found that higher plasma levels of equol, a plant estrogen, were associated with a reduced risk of gastric cancer by analyzing plasma concentrations of equol in 131 cases and 393 controls [146]. Cho et al. also analyzed the interaction effects between single nucleotide polymorphisms (SNPs) involved in the ornithine decarboxylase (ODC)-polyamine pathway, including five genes comprising 30 SNPs, and plasma concentrations of plant estrogens (including equol) through a cohort study. They discovered an increased risk of gastric cancer under low plasma levels of isoflavones but a decreased risk under high plasma levels [147]. These findings suggest that higher concentrations of equol contribute to the prevention and treatment of gastric cancer. Yang et al. found that equol may inhibit proliferation in human gastric cancer MGC-803 cells by inducing G1/G0 arrest and apoptosis through modulation of the AKT pathway [103]. In another study, they demonstrated that equol inhibits progression in gastric cancer by inducing mitochondria-dependent apoptosis via sustained activation of ERK2/1 pathway in MGC-803 cells [104]. There remains limited research on the role of equol specifically for gastric cancer, particularly regarding animal experiments and clinical studies. Further investigation is needed to explore its potential impact within this field.

#### Colorectal cancer

Colorectal cancer (CRC) is the third most common cancer worldwide and the fourth leading cause of cancer-related deaths [148]. Epidemiological studies have found that consuming SI can reduce the risk of colorectal cancer in women by approximately 21% [149]. Shin et al. conducted a case-control study and found an association between intake of soy products or isoflavones and reduced risk of colorectal cancer [150]. A case-control study within the European Prospective Investigation into Cancer and Nutrition analyzed plasma equol concentrations in 809 colon cancer cases and 809 control cases, revealing a negative correlation between plasma equol concentration and colon cancer risk [151]. However, a cohort study involving Swedish women evaluated the relationship between dietary equol intake and colorectal cancer risk, but no significant association was observed. Due to limited number of cases, further confirmation is needed for these results [152]. Despite some contradictory findings, there is more evidence supporting the beneficial effects of equol on colorectal cancer. Zou et al. discovered that racemic equol inhibits proliferation of HCT-15 colon cancer cells through ER binding and antioxidant activity; R-equol inhibits proliferation through antioxidant activity while S-equol has no effect on HCT-15 cell growth [105]. Thus, different isoforms of equol exhibit inconsistent effects on colorectal cancer. Nuclear factor erythroid2-related factor 2 (Nrf2) is a transcription factor involved in regulating oxidative stress response which plays an important role in inducing antioxidant defense mechanisms. It is also considered as an important target for many anti-cancer therapies with antioxidative properties [153]. Cai et al. found that equol can inhibit the growth of colon cancer cells HCT-15 (expressing both ERα and ERβ), LOVO, and SW480 (expressing only ERβ) by upregulating the expression of Nrf2, a key factor involved in antioxidant effects, in vitro [106]. In summary, equol exerts its inhibitory effect on colorectal cancer cell proliferation through binding to ERs and exerting antioxidant activity, providing scientific evidence for the effective role of equol in the prevention and treatment of colorectal cancer.

#### Other non-hormone-dependent tumors

Yu et al. induced a mouse model of lung cancer by subcutaneously injecting mice with ethyl formate, and then intervening with high and low doses of the racemic equol, R-equol, and S-equol diets for 4 months before executing the mice and recording the incidence of lung cancer in each group. Compared to the control group, the experimental groups showed lower incidence rates of lung cancer, with significantly lower rates in the high-dose group compared to the low-dose group. Additionally, except for the low-dose equol group, all other experimental groups exhibited higher levels of Nrf2 protein expression than the control group, with higher levels observed in the high-dose equol group compared to the low-dose equol group [107]. These findings suggest that equol and its enantiomers may inhibit lung cancer development through their antioxidant effects. Jeong et al. investigated the functionality of several polyphenolic compounds (including equol) in epidermal growth factor receptor tyrosine kinase inhibitor (TKI)-resistant non-small cell lung cancer (NSCLC). The results revealed that equol exerted strong inhibitory effects on cell growth in NSCLC HCC827 cell line [108]. Eun et al. studied the impact of combined treatment with tumor necrosis factor-related apoptosis-inducing ligand (TRAIL) and equol on apoptosis in cervical cancer Hela cells. They found that equol enhanced TRAIL-induced apoptosis by activating caspase-3, -8, -9,and Bid cleavage [109]. Cook et al. discovered that higher concentrations of equol inhibited female pancreatic cancer Su 86.86 cell growth but promoted male pancreatic cancer HPAF-11 cell growth [110].

## Discussion

**Fig. 2:**
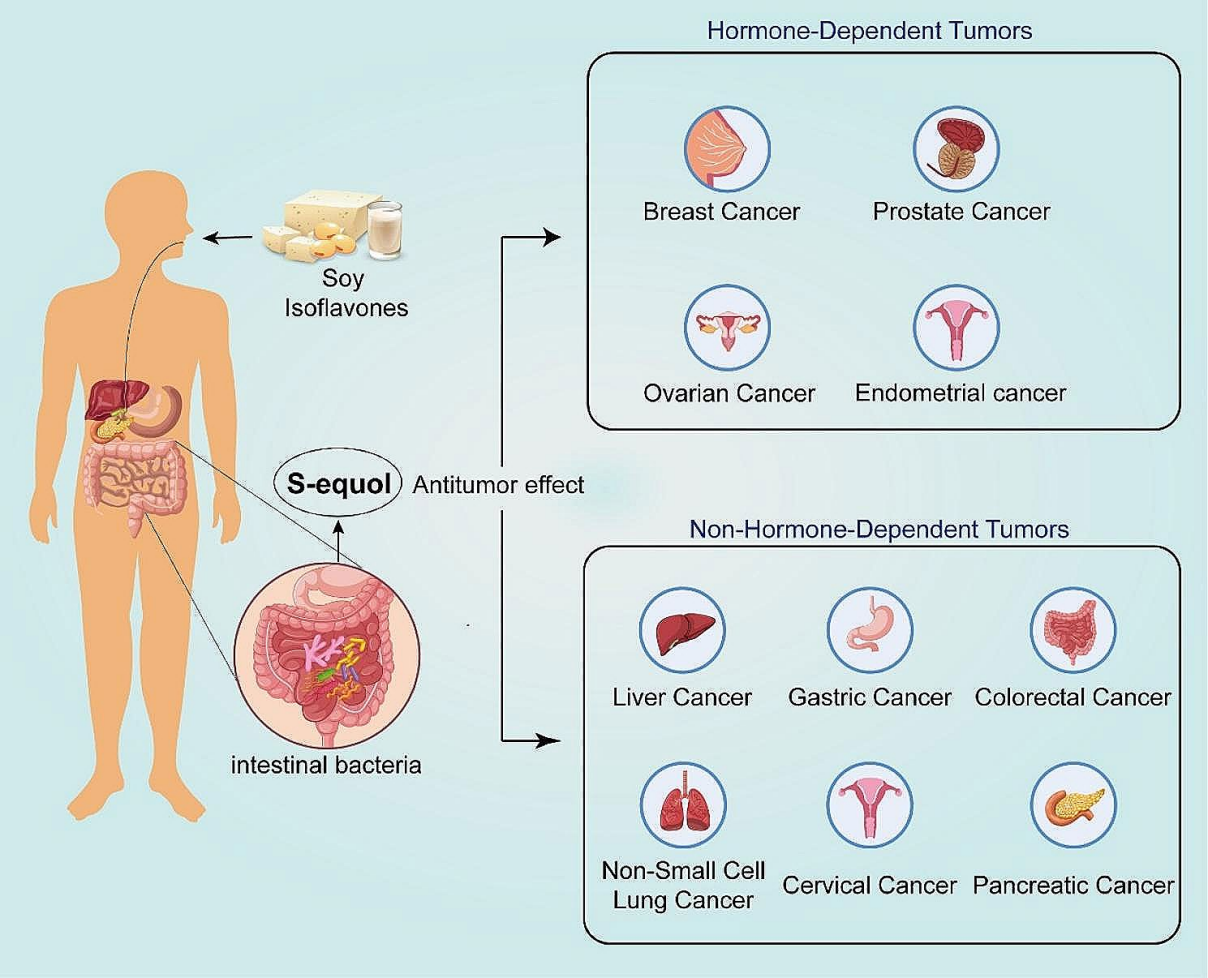
Conversion of dietary isoflavones to equol by human gut microbiota and its antitumor effects schematic illustration

Equol is a metabolite of dietary SI produced through specific intestinal microbial metabolism in the human body. It possesses the highest estrogenic and antioxidant activities among all flavonoid metabolites. Due to its structural similarity to 17-β-estradiol, equol exhibits estrogen-like activity and affinity for both ERα and ERβ. Although endogenous estrogen has positive effects on human health, long-term exposure to exogenous estrogen is widely recognized as a potential risk factor for various types of cancer [154]. Aromatase is an enzyme with significant physiological functions in the human body, capable of catalyzing the conversion of androgens to estrogens. Phytoestrogens can reduce the risk of estrogen-dependent tumors by inhibiting the activity of aromatase, thereby lowering the level of estrogen in the body [155]. According to a study in Japan, equol has been found to promote the excretion of estradiol in female urine and regulate the level of estradiol in the blood [156]. In addition, research by Mahalingam et al. indicates that equol can inhibit the production, action, and metabolism of sex steroid hormones, including endogenous estradiol, testosterone, androstenedione, and progesterone, thereby inhibiting follicular growth and inducing follicular atresia [157]. Integrating these research results, equol can have a positive impact on reducing the risk of various tumors by inhibiting the activity of aromatase. Additionally, equol can increase the activity of endogenous antioxidant enzymes to exert its antioxidant effects [158]. So far, research on equol’s anti-tumor effects has mainly focused on its estrogen-like activity and antioxidant properties; however, further investigation is needed to understand the specific mechanisms by which equol exerts inhibitory effects on different types of tumors. Nevertheless, it is undeniable that equol demonstrates stronger anti-tumor activity than its precursor SI and plays a significant role in preventing and treating various human cancers (as shown in Fig. 2). Numerous epidemiological studies have found a negative correlation between higher concentrations of equol in the body and the risk of multiple cancers [127, 159]. Therefore, it is worth exploring how to increase circulating levels of equol within the body. As widely known, foods rich in SI are the main source of equol production. However, SI primarily exist in an inactive glycoside form that can only be absorbed after being enzymatically converted into bioavailable genistein [160]. Specific intestinal microbiota provide key enzymes for equol metabolism; however, only a few bacterial strains involved in equol metabolism have been reported so far. Moreover, there are variations in how different bacteria metabolize equol. Further research on the role of intestinal microbiota in equol metabolism will help us better understand their crucial contribution to this process. Therefore, it is crucial to increase the intake of soy-based foods and enhance the abundance of individual gut bacteria strains that produce equol. However, the intestinal microbiota in the human body is highly dynamic and constantly changes with diet, age, and antibiotic use [161]. Therefore, the rational use of antibiotics and the development of probiotics containing equol-metabolizing bacterial strains would be beneficial in preventing and treating related diseases by altering the composition of the intestinal microbiota. Additionally, it remains unclear whether supplementing equol-producing bacteria or fecal microbiota transplantation can induce a transition from non-equol producers to equol producers or if an individual’s ability to produce equol will change over time. These are all areas worth investigating and paying attention to in future research.

Early epidemiological studies reported conflicting effects of equol on certain types of cancer. For instance, a prospective cohort study conducted in Europe found that higher levels of equol in serum were associated with an increased risk of breast cancer [162]. This discrepancy can largely be attributed to the fact that previous research did not differentiate between equol producers and non-producers. While numerous studies have demonstrated the potential of equol in effectively preventing various cancers, there is also limited evidence suggesting that equol may promote the progression of non-hormone-dependent breast cancer cells [88, 122]. The contradictory role of equol in tumor research can be explained by several factors. Firstly, these inconsistent results may be influenced by the concentration at which equol exerts its anti-tumor effects, as the effective concentration range for its anti-cancer properties remains unclear. Hatono et al.‘s study shed light on this phenomenon by revealing that equol exhibits dual actions on tumor growth in estrogen receptor-positive breast cancer cell lines: promoting cell growth at low concentrations while exerting anti-tumor effects at high concentrations [88]. Secondly, one of the most well-known biological activities of equol is its estrogen-like action. It can bind to ERs and act as a weak estrogen or competitively bind to ERs on cell nuclei instead of estradiols, thereby exerting an anti-estrogenic effect and maintaining stable hormone levels within the body [113]. However, ERs are distributed unevenly throughout the human body, and both isoforms S-equol and R-equol exhibit different affinities for these two types of receptors. Consequently, the effects produced by equol may vary across different tissues. Existing reports provide more evidence supporting the anticancer activity rather than carcinogenic activity of equol. So far, there is still limited research on equol in terms of its presence in the body and clinical studies. Both epidemiological research and in vitro experiments have certain limitations. The exact mechanism of action for equol’s effective concentration range and its role in various types of tumors are not yet clear. Further studies are needed to explore the effective concentration range of equol, its effects in tumors with different ER expressions, as well as the corresponding actions and mechanisms of two different isomers. This will provide a more reliable theoretical basis for the rational use of equol in various diseases. Although it has been proven that equol can effectively prevent multiple cancers and can be used synergistically with various chemotherapy drugs to exert anti-tumor effects, further research and exploration are still needed on how to safely apply equol to disease prevention and clinical treatment fields.

## Data Availability

No datasets were generated or analysed during the current study.
